# Supplementary material for: MetaRibo-Seq measures translation in microbiomes
Source: Nat Commun. 2020 Jun 29;11:3268. doi: 10.1038/s41467-020-17081-z (PMC7324362; doi:10.1038/s41467-020-17081-z)
Supplement: Supplementary file 10 — Supplementary Data 7 [file 41467_2020_17081_MOESM10_ESM.zip › File2/Confidence_High_Taxonomy/141324_out.krona.html]

Javascript must be enabled to view this page.

members
magnitude
magnitudeUnassigned
count
unassigned
taxon
rank

141324\_out

18

superkingdom
2
18

phylum
1239
18

class
91061
1

order
186826
1

1300
family
1

genus
1301
1

1

SRS098640\_contig\_number\_6587
species
1305

2292887

SRS144603\_contig\_number\_contig-100\_10426.54072
species
1

16
186801
class

3
16

SRS1055067\_contig\_number\_contig-100\_30913.71855SRS143780\_contig\_number\_1576SRS148253\_contig\_number\_contig-100\_7641.53666
order
186802

family
186803
4

2292271

SRS075773\_contig\_number\_contig-100\_3095.84356
species
1

1
genus
841

360807

SRS104975\_contig\_number\_contig-100\_118.119
species
1

2
genus
43996

43997
species

SRS063603\_contig\_number\_contig-100\_13434.49967SRS097918\_contig\_number\_contig-100\_5276.5277
2

2
31979
family

1485
genus
2

1637498

SRS015782\_contig\_number\_contig-100\_4587.138985SRS052078\_contig\_number\_1051
species
2

7
family
541000


SRS055982\_contig\_number\_contig-100\_5833.47159SRS144297\_contig\_number\_contig-100\_17989.60117
genus
1263
2
7

2292069

SRS024388\_contig\_number\_1734
species
1

1
species

SRS893380\_contig\_number\_contig-100\_277.278
2292251

1
2293189

SRS046712\_contig\_number\_contig-100\_5918.26737
species


SRS018623\_contig\_number\_contig-100\_28595.76118
species
2292071
1

1

SRS016018\_contig\_number\_7741
species
2293218
